# Intermittent Fasting Ameliorated High-Fat Diet-Induced Memory Impairment in Rats via Reducing Oxidative Stress and Glial Fibrillary Acidic Protein Expression in Brain

**DOI:** 10.3390/nu13010010

**Published:** 2020-12-22

**Authors:** Suzan M. Hazzaa, Mabrouk A. Abd Eldaim, Amira A. Fouda, Asmaa Shams El Dein Mohamed, Mohamed Mohamed Soliman, Eman I. Elgizawy

**Affiliations:** 1 Medical Physiology Department, Faculty of Medicine, Menoufia University, Shebeen Elkom 32511, Egypt; suzanhazzaa@med.menofia.edu.eg (S.M.H.); eman.elgizawi@med.menofia.edu.eg (E.I.E.); 2 Department of Biochemistry and Chemistry of Nutrition, Faculty of Veterinary, Medicine, Menoufia University, Shebeen Elkom 32511, Egypt; 3 Pathology Department, Faculty of Medicine, Menoufia University, Shebeen Elkom 32511, Egypt; aamf8296@gmail.com (A.A.F.); asmaashams@rocketmail.com (A.S.E.D.M.); 4 Clinical Laboratory Sciences Department, Turabah University College, Taif University, Taif 21995, Saudi Arabia; mmsoliman@tu.edu.sa; 5 Biochemistry Department, Faculty of Veterinary Medicine, Benha University, Benha 13736, Egypt

**Keywords:** high fat diet, intermittent fasting, GFAP

## Abstract

Intermittent fasting (IF) plays an important role in the protection against metabolic syndrome-induced memory defects. This study aimed to assess the protective effects of both prophylactic and curative IF against high-fat diet (HFD)-induced memory defects in rats. The control group received a normal diet; the second group received a HFD; the third group was fed a HFD for 12 weeks and subjected to IF during the last four weeks (curative IF); the fourth group was fed a HFD and subjected to IF simultaneously (prophylactic IF). A high-fat diet significantly increased body weight, serum lipids levels, malondialdehyde (MDA) concentration, glial fibrillary acidic protein (GFAP) and H score in brain tissue and altered memory performance. In addition, it significantly decreased reduced glutathione (GSH) concentration in brain tissue and viability and thickness of pyramidal and hippocampus granular cell layers. However, both types of IF significantly decreased body weight, serum lipids, GFAP protein expression and H score and MDA concentration in brain tissue, and improved memory performance, while it significantly increased GSH concentration in brain tissue, viability, and thickness of pyramidal and granular cell layers of the hippocampus. This study indicated that IF ameliorated HFD-induced memory disturbance and brain tissue damage and the prophylactic IF was more potent than curative IF.

## 1. Introduction

Widespread metabolic syndrome (MS) nowadays is a sign of many underlying health problems, which result mainly from consumption of a high-fat diet (HFD) with high energy input [1]. Metabolic syndrome is associated with body fat accumulation, hypertension, dyslipidemia, hyperglycemia and oxidative stress [2]. It seriously affects the brain, behavior, and memory [3] and raises the risk of dementia [4]. Memory impairment in MS appears to be due to the reduction in many factors including the cholinergic system, and signal transduction together with the reduction in hippocampal neuron density [4].

On the other hand, dietary restriction (DR), either by reduced energy intake or intermittent fasting (IF) has been proven to increase the quality and span of the life with reduction of the incidence of age-associated diseases [5]. Dietary restriction and physical exercise have been proven as an effective measure to reduce the risk of cardiovascular disease in obese humans [6]. Additionally, long-term DR has been reported to reduce serum lipid concentrations and arterial blood pressure. Moreover, it has a neuroprotective effect as it can delay neuronal degeneration in Alzheimer’s disease [7]. Initiation of DR early in the adulthood stage has been reported to be the only means of delaying the onset of the age associated diseases. Other studies reported that DR even initiated at late age or for a limited time can also have beneficial effects [8,9]. Dietary restriction has antioxidant and anti-inflammatory properties [10]. Intermittent fasting in animal models is a DR regimen in which food is allowed but only every other day [11]. It can prevent neuro inflammation and oxidative stress [6].

Thus, it is important to change the current feeding habits and to find a new easy applicable strategy to prevent these health hazards. Intermittent fasting is a new dietary restriction method that is proven to boost body metabolism [12], decrease body fat, and body weight [13], as well as cognitive impairment [14]. The current study aimed to assess the potential effects of both curative and prophylactic intermittent fasting against high-fat diet-induced metabolic syndrome in rats.

## 2. Material and Methods

The experimental protocol was approved by the local ethical committee of the Faculty of Medicine, Menoufia University with approval code 279/018 following the Guide for the Care and Use of Laboratory Animals (eighth edition, National Academies Press) [15].

### 2.1. Animals

Forty adult male Wister albino rats matched for weight and age between 150 and 200 g were used in this experiment. During the study, each 2 rats were kept in one cage at normal room temperature, humidity and normal light/dark cycle.

### 2.2. Experimental Design

Rats were randomly assigned into four experimental groups, 10 rats each.

Control group: rats were fed standard rat corn-based chow (Table 1) for 12 weeks [16].

High-fat diet (HFD) group: rats were fed HFD (Table 2) for 12 weeks [17].

Curative intermittent fasting group (cur. IF): rats were fed HFD for 12 weeks and subjected to IF during the last 4 weeks alternating with HFD diet [11].

Prophylactic intermittent fasting group (pro. IF): rats were subjected to IF for 24 h and fed HFD in the other day from the beginning of the experiment until its end.

**Figure 1:**
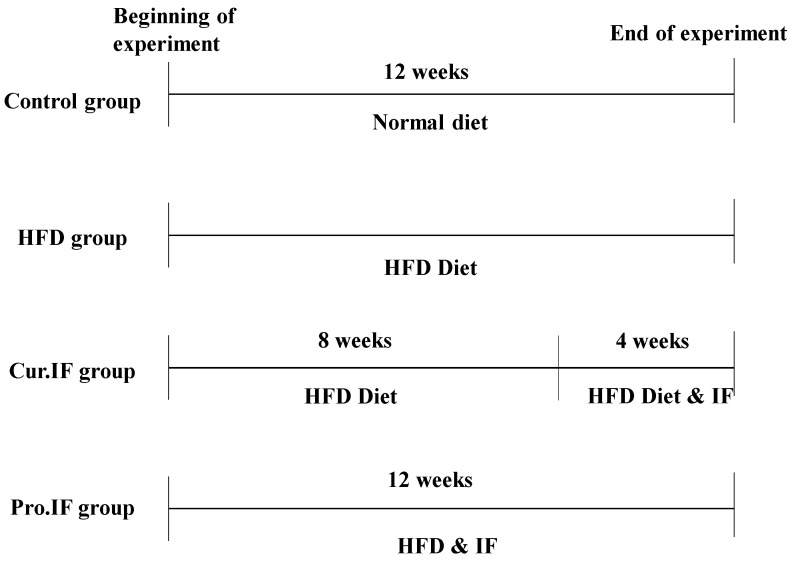
Diagram representing the experimental design. Rats were fed either basal (normal control group) diet; HFD; HFD and subjected to intermittent fasting (pro. IF) for 12 weeks, or HFD for 12 weeks and subjected to intermittent fasting (cur. IF) alternating with HFD during last 4 weeks. HFD means high-fat diet; pro. IF means prophylactic intermittent fasting while cur. IF means curative intermittent fasting.

The experimental design is illustrated below (Figure 1).

### 2.3. Sampling

Rat body weights were recorded at the start of the experiment and at the end of the 4th and 8th weeks and at the end of the experiment (after 12 weeks from the beginning of the experiment) and the behavioral tests were performed as described below. Animals then were fasted overnight, and blood samples were collected, and sera samples were separated and used for measurements of fasting serum lipid profile. Then, rats were sacrificed by cervical decapitation and brains were removed and divided into two parts. One part was kept at −80 °C and used for estimation of malondialdehyde (MDA) and reduced glutathione (GSH) concentrations. The other half was kept in 10% neutral formalin and used for histopathological and immunohistochemical investigations.

### 2.4. Assessment of Behavioral Responses

All behavioral tests were performed between 9:00 a.m. and 2:00 p.m. in a calm observation room with normal day light. Rats were first adapted for 1 h before the beginning of the tests in the observation room. All tests were observed through video camera (Samsung ST93 Digital Camera, Suwon, South Korea). The equipment was cleaned with 70% ethanol to avoid odor cues for animals [18].

### 2.5. Assessment of Motor Function

#### Open Field Test

A wooden arena (100 × 100 × 60 cm height, with brown walls and floor) was divided into equal 25 squares. Rat was put in the center of the open field for 15 min and freely allowed to explore it [18]. The latency to move from the center was calculated, and the total distance moved by meter (m) was measured by calculating the numbers of crossed squares. The frequency of grooming was counted manually, time spent in the inner or outer zones also was calculated. After each test, the field was cleaned with 90% alcohol solution. 

### 2.6. Assessment of Short-Term Spatial Memory

#### Y-Maze

The Y-maze consisted of three arms, each one 50 cm long, 10 cm wide and 20 cm high. It was used to evaluate the short-term spatial memory [19]. The Y-maze was made of wood and elevated 50 cm above the floor. This task was based on the innate behavior of animals to explore new areas. It consisted of two trials; a training phase and a test phase, each of them consisted of 5 min and was separated by a 90 min interval. During the training phase, the novel arm was blocked by a removable door then the rat was placed at the beginning of the start arm and left for 5 min to explore two arms. During the test phase, the “novel” arm was opened, and the rat could explore the available arms. Rats were observed by video camera. The number of the entries into the different arms and the time that rat spent in these arms were recorded.

### 2.7. Biochemical Investigations

Serum total cholesterol, triacylglycerol (TG) and high-density lipoprotein (HDL) levels were estimated by using an automatic analyzer 902 (Hitachi, Munich, Germany) and commercial kits (Bio-Med diagnostic, Cairo, Egypt). Serum levels of low-density lipoprotein (LDL) and very-low-density lipoprotein (VLDL) levels were analyzed according to the method of DeLong [20]. Serum level of VLDL was calculated by dividing serum level of TG by five [21]. Serum levels of low-density lipoprotein (LDL) were obtained by using the following equation [20]:

LDL − C (mmol/L) = TC − HDL − C − TG/2.2(1)

### 2.8. Measurement of Brain Tissues Malondialdehyde and Reduced Glutathione Concentrations

Brain tissues were homogenized in normal saline solution (1:9 *w*/*v*). The homogenate was centrifuged at 1800× *g*/min for 10 min. The supernatant was used for the measurements of lipid peroxidation and antioxidant enzyme activity via malondialdehyde (MDA). The concentration of malondialdehyde was quantified spectrophotometrically by using commercial kits (Biodiagnostic Company, Cairo, Egypt) according to [22]. The reduced glutathione (GSH) concentration was measured by using kits purchased from Biodiagnostic Company, Egypt, according to [23].

### 2.9. Histopathological Examination

Brain tissue samples of all groups were rapidly excised, cut into small pieces, and fixed in 10% neutral formalin. Then, tissue sections were processed and stained by hematoxylin and eosin stain according to [24].

### 2.10. Immunohistochemical Investigations

Glial fibrillary acidic protein (GFAP) was detected by using avidin–biotin complex (ABC) immunoperoxidase technique. After blocking the endogenous peroxidase, brain sections were incubated with anti-GFAP primary antibody at (1:100 dilution) for 20 min at room temperature. The primary GFAP antibody was mouse monoclonal antibody, (GFAP) Ab-1 (Clone GA-5), specific to the astrocytes obtained from Lab Vision Corporation, Medico Co., Egypt (Thermo Fisher, UK, Cat. #MS-280-R7). Then, the slides were incubated with the secondary anti-mouse antibody universal kits for 30 min in a humid chamber at room temperature after washing with diluted phosphate-buffered saline. All sections were stained by incubation with 3,3’-diaminobenzidine (DAB), a substrate chromogen, for 5–10 min resulting in brown-colored precipitate at the antigen sites. The Mayer’s hematoxylin was used as a counter stain. Positive control was Cellosaurus cell line (IMR5) while for negative controls, incubation was without the primary antibody. The positive reactivity of GFAP was exhibited as different grades of reactivity (i.e., weak, moderate and strong), according to the intensity of staining. Positive reactivity was indicated by a brown-colored reaction [25].

H scoring: staining of the membrane was scored into four categories: 0 means no staining, 1 + means light staining visible only at high magnification, 2 + means intermediate staining, while 3 + means dark staining of linear membrane visible even at low magnification. The percentage of cells at different staining intensities was determined by visual assessment, with the score calculated using the formula 1 × (% of 1 + cells) + 2 × (% of 2 + cells) + 3 × (% of 3 + cells) [26]. Images were captured by using a colored video camera (Panasonic Color CCTV camera, Matsushita Communication, Industrial Co. Ltd., Tokyo, Japan) fixed on a light microscope (Olympus BX-40, Olympus Optical Co. Ltd., Tokyo, Japan). Images were taken at 400× magnification and 2.6 zoom. Photomicrographs were analyzed by using Software Image J program, a public domain image processing and analysis program (U.S. National Institutes of Health, Bethesda, MD, USA) (http://rsb.info.nih.gov/ij/) [27].

### 2.11. Statistical Analysis

Our data were expressed as mean ± standard error of the mean (SEM). The statistical analysis was carried out by using SPSS version 22 (IBM Corp., Armonk, NY, USA). Statistical analysis for behavior tests on open field and Y-mazes were performed by using Kruskal–Wallis and Mann–Whitney tests using all data sets to ensure normal distribution (*p*  >  0.5). Other results were analyzed by using one-way ANOVA (analysis of variance), followed by (Tukey’s) post hoc test for determination of significance of differences among groups. Differences were considered significant at *p* < 0.05.

## 3. Results

### 3.1. Intermittent Fasting Ameliorated HFD Increased Rats’ Body Weight

**Figure 2:**
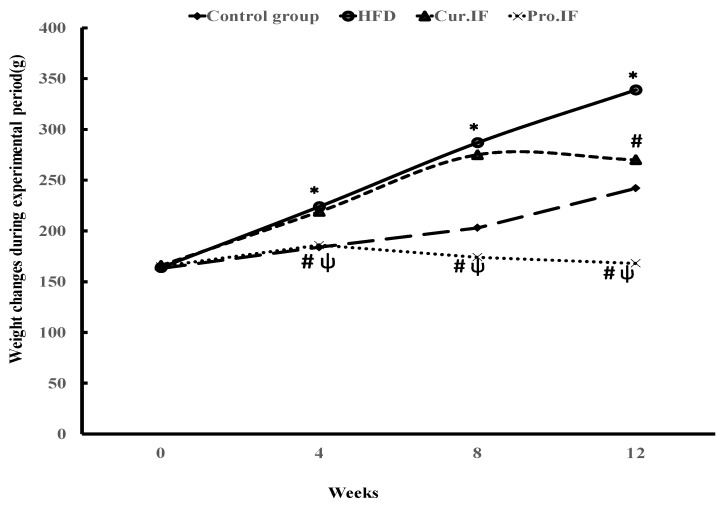
Effect of HFD and/or intermittent fasting on rats’ body weights: Rats were fed either (a) basal (normal control group) diet; (b) HFD; (c) pro. IF; rats were subjected to both IF and HFD for 12 weeks; or (d) cur. IF; rats were subjected to HFD for 8 weeks then IF simultaneously with HFD during last 4 weeks. Rats’ body weights were recorded after 4, 8 and 12 weeks from the beginning of the experiment. In all studied groups * HFD significant vis. control group, # pro. and cur. IF significant vis. HFD group, ψ pro. IF significant vise cur. IF.

Figure 2 showed rats’ body weights of all experimental groups. High-fat diet significantly (*p* < 0.001) increased rats’ body weight after 4, 8 and 12 weeks from the beginning of the experiment compared with the control rats fed standard diet. However, pro. IF significantly (*p* < 0.001) decreased rats’ body weights after 4, 8 and 12 weeks from the beginning of the experiment compared to both HFD and cur. IF groups. Whereas cur. IF significantly (*p* < 0.001) decreased rats’ body weight only after 12 weeks compared with HFD group.

### 3.2. Intermittent Fasting Improved the Behaviors of HFD Fed Rats

#### 3.2.1. Open Field

**Figure 3:**
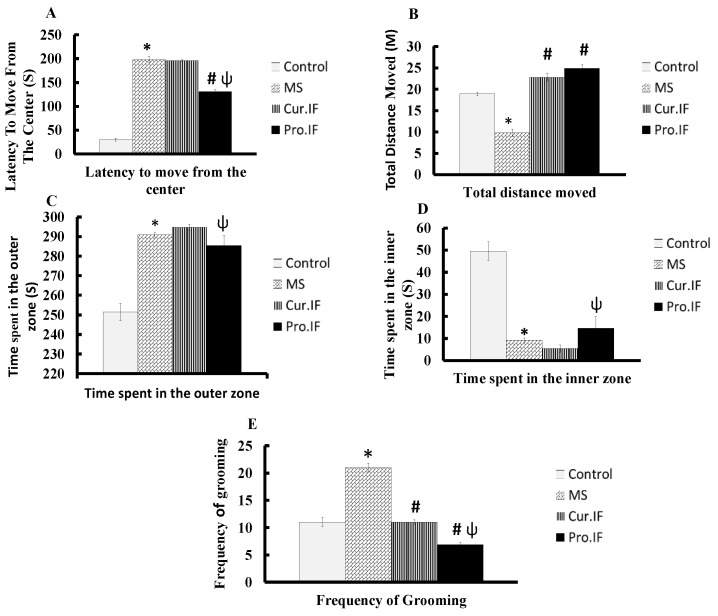
Effect of HFD and/or pro. and cur. IF on (**A**) latency to move from the center, (**B**) total distance moved, (**C**) time spent in the outer zone, (**D**) time spent in the inner zone, and (**E**) frequency of grooming in the open field test. Data are expressed as mean ± SEM (*n* = 10). * HFD significant vis. control group, # pro. and cur. IF significant vis. HFD group, ψ pro. IF significant vise cur. IF.

Figure 3 showed the effects of HFD and/or IF on rats’ motor functions and behavioral changes. High-fat diet significantly increased (*p* < 0.001) the length of time needed to move from the center of open field, time spent in the outer zone and the frequency of grooming compared with the control group. On the other hand, it significantly (*p* < 0.001) decreased total distance moved in open field and time spent in inner zone. Cur. IF significantly (*p* < 0.001) increased total distance moved while significantly (*p* < 0.001) decreased frequency of grooming compared with HFD group. Additionally, pro. IF significantly (*p* < 0.001) decreased latency to move from the center and the frequency of grooming while it significantly (*p* < 0.001) increased the total distance moved compared with HFD group. In addition, it significantly (*p* < 0.001) decreased latency to move from the center, time spent in the outer zone and the frequency of grooming with significant (*p* < 0.001) increase in the time spent in the inner zone compared with cur. IF group (Figure 3).

#### 3.2.2. Y-Maze

**Figure 4:**
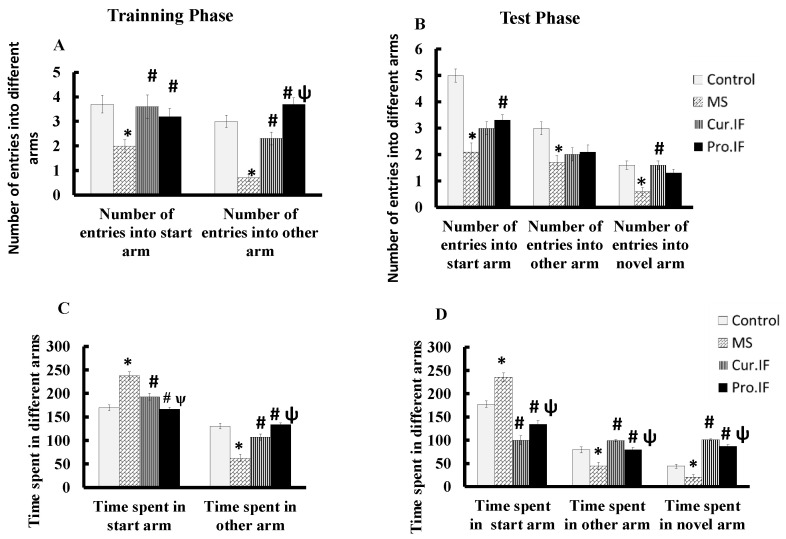
Effect of HFD and/or pro. and cur. IF on the short-term spatial memory tested by Y-maze in different studied groups: (**A**) the number of entries in the training phase; (**B**) the number of entries in the test phase; (**C**) the time spent in the training phase; (**D**) the time spent in the test phase. Data are expressed as mean ± SEM. (*n* = 10). * HFD significant vis. control group, # pro. and cur. IF significant vis. HFD group, ψ pro. IF significant vise cur. IF.

Figure 4 shows the effect of HFD and/or IF on rats’ behavior and short-term memory using Y-maze. Feeding rats HFD significantly (*p* < 0.001) decreased number of entries into all arms in training and test phases and the time spent in the other arm and novel arm in both phases compared with the control group. However, IF cur. and pro. IF significantly (*p* < 0.001) increased the number of the entries in both arms while it significantly (*p* < 0.001) decreased time spent in the start arm in the training phase compared with HFD group. Additionally, IF significantly (*p* < 0.001) increased the time spent in the novel arm in test phase compared with HFD group (Figure 4).

### 3.3. Intermittent Fasting Ameliorated HFD Altered Serum Lipid Profile in Rats

Table 3 shows that feeding rats HFD significantly (*p* < 0.001) increased serum cholesterol, triglycerides, LDL and VLDL levels while it significantly (*p* < 0.001) decreased serum HDL level compared with the control rats fed standard diet. Both the cur. and pro. IF significantly (*p* < 0.001) decreased serum cholesterol, triglycerides, LDL and VLDL levels compared with HFD group. In addition, pro. IF significantly (*p* < 0.001) increased serum HDL levels compared with those of HFD group. Moreover, pro. IF significantly (*p* < 0.001) decreased serum levels of cholesterol, triglycerides, LDL and VLDL while it significantly (*p* < 0.001) increased serum HDL level compared with curative IF group (Table 3).

Data are expressed as mean ± SEM (*n* = 10); * HFD significant vis. control group, # pro. and cur. IF significant vis. HFD group, ψ pro. IF significant vise cur. IF.

### 3.4. Intermittent Fasting-Modulated HFD Altered Malondialdehyde and Reduced Glutathione Concentrations in the Brain Tissues

**Figure 5:**
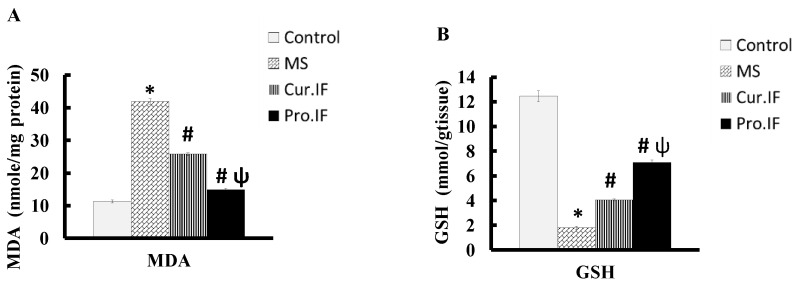
Effect of HFD and/or pro. and cur. IF on the brain tissue contents of MDA (**A**) and GSH (**B**) in all studied groups * HFD significant vis. control group, # pro. and cur. IF significant vis. HFD group, ψ pro. IF significant vise cur. IF.

Feeding rats HFD significantly (*p* < 0.001) increased brain tissue MDA concentration while it significantly (*p* < 0.001) decreased brain tissue GSH concentration compared with the control group. However, both the cur. and pro. IF significantly (*p* < 0.001) decreased brain tissue MDA concentration while it significantly (*p* < 0.001) increased brain tissue GSH concentration compared with HFD group. Moreover, pro. IF significantly (*p* < 0.001) reduced brain tissue contents of MDA while it significantly (*p* < 0.001) increased brain tissue content of GSH compared with cur. IF group (Figure 5A,B).

### 3.5. Intermittent Fasting-Ameliorated HFD Induced Histopathological Changes in Brain Tissues of Rats

**Figure 6:**
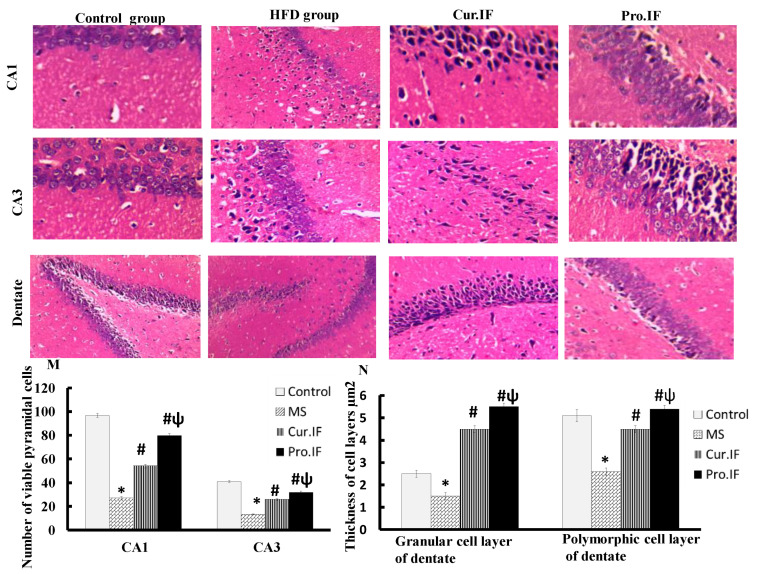
Effect of HFD and/or pro. and cur. IF on hippocampal structure. The figure showed normal morphology of CA1, CA3 and dentate gyrus in the control group with normal viable pyramidal cells, granular cells, absent apoptosis and normal blood vessels. The HFD group showed scattered degenerations, decrease in the viable pyramidal cells in both CA1 and CA3 areas of the hippocampus (**M**) together with decrease in the thickness of granular cells in dentate gyrus (**N**). The cur. IF group showed multiple degenerated cells, mild apoptosis while pro.IF group showed marked increase in the viable pyramidal cells with scattered degeneration in CA1 and CA3 regions dentate gyrus showed increase in thickness of its layers in both groups. * HFD significant vis. control group, # pro. and cur. IF significant vis. HFD group, ψ pro. IF significant vise cur. IF (**N**).

Figure 6 showed normal structure and morphology of the hippocampus of the control group with normal viability and thickness of the pyramidal cell layer and normal blood vessels with no apoptosis in both CA1 and CA3 areas. Dentate gyrus of the same group showed normal granular and polymorphic cell layers. Brain tissue sections of HFD group showed multiple degenerated cells, marked apoptosis with a decrease in the thickness of all layers of the hippocampus. Intermittent fasting significantly increased the thickness of all layers of the hippocampus while it decreased the number of apoptotic and degenerated cells compared with HFD group. The pro. IF group showed more significant increase in the thickness of all layers of the hippocampus with increase in the viability of pyramidal cell layer compared with cur. IF (Figure 6M,N).

### 3.6. Intermittent Fasting Reduced HFD Increased GFAP Protein Expression in Brain Tissues of Rats

**Figure 7:**
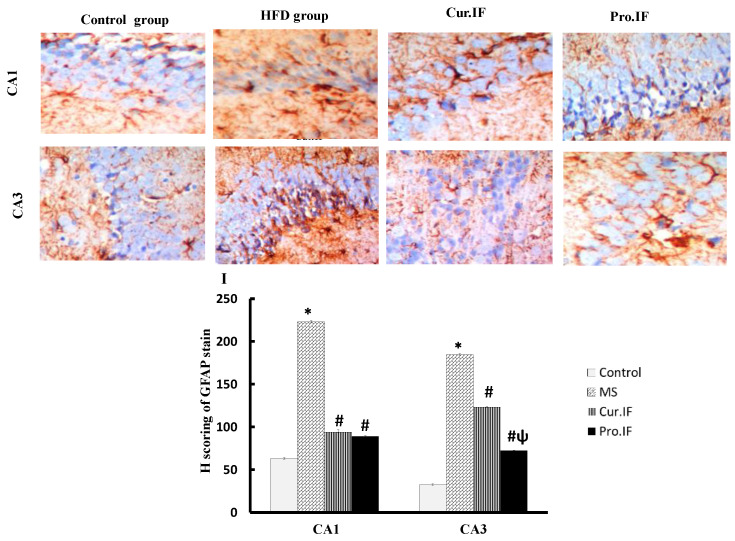
Effect of HFD and/or pro. and cur. IF on GFAP protein expression in the hippocampal sections in all studied groups. The figure shows significant increase in GFAP protein expression in CA1 and CA3 of HFD group. The cur. IF and pro. IF groups showed decreased GFAP protein expression in the same areas. The histogram (**I**) declares the changes in H score in these areas. * HFD significant vis. control group, # pro. and cur. IF significant vis. HFD group, ψ pro. IF significant vise cur. IF.

Figure 7 illustrates that the hippocampal cells of the HFD group showed strong GFAP protein expression compared with that of the control group. However, hippocampus of both cur. and pro. IF groups showed weak and localized protein expression of GFAP compared with that of the control and HFD groups. Figure 7I shows that the H score was significantly increased in CA1 and CA3 areas of HFD group. Cur. and pro. IF groups significantly decreased H score in the same areas compared with HFD group. The pro. IF caused significant decrease in H score in CA3 area compared with that of the cur. IF group.

## 4. Discussion

The results of the current study revealed that the consumption of HFD increased rats’ body weight while it decreased the short term-memory with abnormal behavior, dyslipidemia and oxidative stress in brain tissues. The HFD increased body weight was in line with the findings of Abd Eldaim et al., 2018 and Orabi et al., 2020 [28,29]. This may be due to HFD increasing food intake with excess energy intake and adiposity buildup as HFD creates more fat storage than fat oxidation in muscle. High-fat diet-induced abnormal behavior in rats was represented by decreased total distance moved in the open field and the time spent in the inner zone, increased the latency to move from the center, the time spent in the outer zone and the frequency of grooming. These findings may be due to HFD decreasing physical activity and physical efficacy with anxiety in rats [30]. In addition, HFD affected the normal behavior of rats in the Y maze, which is based on the natural preference of rodents to explore the novel environment rather than the previously known one. It increased the time spent in the start arm and decreased the frequency of entry and the total time spent in the novel arm. These data can be attributed to oxidative stress indicated by high level of MDA with low level of GSH in the brain tissues. Oxidative stress contributes to many neurodegenerative diseases and brain damage, and induces cell injury with impaired learning and memory. Additionally, one study relates Alzheimer’s disease to consumption of HFD [31] while other studies attribute this cognitive impairment to other factors such as impaired glucoregulation [32], increased brain inflammation and alteration in blood brain barrier permeability [33]. It has been known that consumption of HFD is associated with significant weight gain and chronic low levels of inflammation together with brain insulin resistance with loss of synaptic plasticity [34]. Previous epidemiological data revealed an association between obesity, high-fat intake and cognitive dysfunctions [35]. This is in line with data revealed by Valladolid, who found that HFD impaired learning trials and spacial memory at the level of the hippocampus [36]. The effect of HFD occurs even after a short period of intake, as the memory deficit was detected after only one week [37]. Tran and Westbrook indicated that the cognitive dysfunction is reversible after stopping the HFD as they speculated that stopping HFD decreases the inflammatory condition induced by HFD and obesity [38]. Moreover, our data indicated hippocampal pyramidal cell affection with decreased viability, thickness and increased apoptosis with significant increase in the GFAP immunostaining in the HFD group, which might be attributable to inflammation of astrocytes due to consumption of HFD, which stimulates astrogliosis, as it has been shown that HFD causes hippocampal dysfunction and affects short-term memory [39].

Furthermore, the current study indicated that intermittent fasting improved brain structure and function by decreasing oxidative stress. This finding also can be explained by a significant decrease in oxidative stress in the brain tissue, which was indicated by significant decrease in MDA contents with significant increase in GSH contents in brain tissues. Caloric restriction by IF improves the brain redox state and increases GSH level as mentioned by Rebrin et al., 2007 [40], who found that 40% reduction in caloric intake improves oxidative stress in different brain areas in aged mice. Additionally, IF has a neuroprotective effect and increases the resistance of the hippocampus to excitotoxic stress, which may be due to mitochondrial reprogramming that decreases oxidant production [14]. According to Hu et al., short-term IF is a neuroprotective that can reduce the redox state and the neuro-inflammation. It has also been reported that post-operative IF reduces the concentration of MDA in brain tissues and increased GSH concentration in brain tissues [6]. Moreover, the current study indicated more significant decrease in oxidative stress and body weight with better performance of rats in behavioral tests of pro. IF compared to cur. IF. This may be due to the simultaneous neuroprotective action of IF that decreases the deleterious effect of HFD on brain oxidative stress and body weight. This was clear in the histological and immunohistochemical staining that showed significant increases in the thickness and viability of pyramidal cells and granular cells in the hippocampus. There were also significant decreases in the GFAP stain and H score in both IF groups. The pro. IF caused a more significant increase in the viability and thickness of pyramidal and granular cell layers, with significant decrease in H score in the CA3 area compared to the cur. IF group.

Finally, the findings of the current study indicated that HFD induced significant dyslipidemia represented by significant increase in serum levels of TC, TG and LDL with significant decrease in HDL. This hyper-lipidemia might result from HFD-induced oxidative stress. Both pro. and cur. IF significantly decreased fasting serum lipids levels. These findings may be due to the fact that dietary restriction of calories can improve lipid profile parameters. It increases the activity of the lipoprotein lipase, resulting in increases of triglyceride clearance in the blood vessels. The activated LPL also increase the catabolism of lipoproteins rich in triglycerides, resulting in the transfer of esters, apoproteins and phospholipids to form HDL [41]. Moreover, IF decreases caloric intake, so the production of apolipoprotein A-1 may be optimal with subsequent increase in HDL concentrations in serum [42]. Our data were in line with Marbut et al. (2005) [43], as there was significant decrease in serum cholesterol, TGs and LDL levels with significant increase in HDL levels after IF for one month. Nurmasitoh et al. [44] also found higher HDL levels in rats subjected to IF.

## 5. Conclusions

Consumption of HFD increased rats’ body weight while it decreased short term-memory with abnormal behavior, dyslipidemia and oxidative stress in brain tissues. In contrast, intermittent fasting significantly decreased body weight and oxidative stress, as well as improving short term-memory, behavior and dyslipidemia in HFD-fed rats. The pro. IF was more potent than cur. IF as it gave the brain a longer period of neuro-protection and resistance to oxidative stress and inflammation.

## Figures and Tables

**Table 1:** Chemical composition of basal standard diet.

Diet Constituents	Control Group
Fat	7–10%
Carbohydrates	68–70%
Protein	18–20%
Vitamins and minerals	1–2%
Kcal/100 g/day	341

**Table 2:** Chemical composition of high-fat diet.

Diet Constituents	HFD
Fat	30%
Carbohydrates	50–52%
Protein	18–20%
Vitamins and minerals	1–2%
Kcal/100 g/day	530

**Table 3:** Effects of HFD and IF on serum lipid profile of all experimental groups.

Metabolite	Control	HFD	HFD & Cur IF	HFD & Pro IF
Cholesterol (mg/dL)	119.60 ± 1.88	277.10 ± 1.84 *	174.50 ± 2.37 ^#^	129 ± 1.24 ^#ψ^
Triglycerides (mg/dL)	122 ± 3.54	203.00 ± 7.20 *	174.70 ± 2.07 ^#^	134.70 ± 2.54 ^#ψ^
LDL (mg/dL)	43.60 ± 3.77	209.78 ± 2.22 *	109.06 ± 2.18 ^#^	70.16 ± 1.94 ^#ψ^
HDL(mg/dL)	51.60 ± 2.52	26.70 ± 0.97 *	31.50 ± 1.82	39.00 ± 1.52 ^#ψ^
VLDL (mg/dL)	24.40 ± 0.70	40.62 ± 0.63 *	34.94 ± 0.41 ^#^	26.94 ± 0.50 ^#ψ^

Data are expressed as mean ± SEM (*n* = 10); * HFD significant vis. control group, # pro. and cur. IF significant vis. HFD group, ψ pro. IF significant vise cur. IF.

## Data Availability

The data presented in this study are available on request from the corresponding author.
